# Interactions Between Drought and Plant Genotype Change Epidemiological Traits of *Cauliflower mosaic virus*

**DOI:** 10.3389/fpls.2018.00703

**Published:** 2018-05-24

**Authors:** Sandy E. Bergès, Denis Vile, Cecilia Vazquez-Rovere, Stéphane Blanc, Michel Yvon, Alexis Bédiée, Gaëlle Rolland, Myriam Dauzat, Manuella van Munster

**Affiliations:** ^1^BGPI, CIRAD, INRA, Montpellier SupAgro, Université de Montpellier, Montpellier, France; ^2^LEPSE, INRA, Montpellier SupAgro, Université de Montpellier, Montpellier, France; ^3^LABINTEX Europe, Instituto Nacional de Tecnología Agropecuária, Montpellier, France

**Keywords:** plant growth traits, viral load, viral transmission, virulence, tolerance, plant–virus interactions, water deficit

## Abstract

Plants suffer from a broad range of abiotic and biotic stresses that do not occur in isolation but often simultaneously. Productivity of natural and agricultural systems is frequently constrained by water limitation, and the frequency and duration of drought periods will likely increase due to global climate change. In addition, phytoviruses represent highly prevalent biotic threat in wild and cultivated plant species. Several hints support a modification of epidemiological parameters of plant viruses in response to environmental changes but a clear quantification of plant–virus interactions under abiotic stresses is still lacking. Here we report the effects of a water deficit on epidemiological parameters of *Cauliflower mosaic virus* (CaMV), a non-circulative virus transmitted by aphid vectors, in nine natural accessions of *Arabidopsis thaliana* with known contrasted responses to water deficit. Plant growth-related traits and virus epidemiological parameters were evaluated in PHENOPSIS, an automated high throughput phenotyping platform. Water deficit had contrasted effects on CaMV transmission rate and viral load among *A. thaliana* accessions. Under well-watered conditions, transmission rate tended to increase with viral load and with CaMV virulence across accessions. Under water deficit, transmission rate and virulence were negatively correlated. Changes in the rate of transmission under water deficit were not related to changes in viral load. Our results support the idea that optimal virulence of a given virus, as hypothesized under the transmission-virulence trade-off, is highly dependent on the environment and growth traits of the host.

## Introduction

Under field conditions, plants are exposed to various biotic and abiotic stresses that can impact their performance and their population dynamics and ecology (Suzuki et al., 2014; Pandey et al., 2015; Prasch and Sonnewald, 2015; Ramegowda and Senthil-Kumar, 2015; Aou-ouad et al., 2017). In particular, soil water deficit (WD) and plant virus diseases are major abiotic and biotic constraints impacting plant physiology and growth as well as agricultural productivity worldwide (Zhang and Sonnewald, 2017). The relationships between host plants and viruses appear more complex than previously described, particularly regarding the role of perturbing environmental factors. To date, environmental conditions and plant physiological mechanisms that can lead to contrasted relationship with viruses, from mutualism to increased pathogenicity, remain poorly studied (Roossinck, 2015). Few recent investigations combining abiotic and biotic stresses clearly demonstrate that in addition to factors related to the virus and plant genotype the fate of plant–virus interactions also depends on the abiotic environment (Prasch and Sonnewald, 2013; Ramegowda and Senthil-Kumar, 2015; Fraile and García-Arenal, 2016; Aguilar et al., 2017; Carr, 2017). A growing body of data is uncovering the intimate entanglement of the plant physiological pathways involved in responses to various abiotic stresses and in defense against pathogens and herbivores (Bostock, 2005; Pandey et al., 2015; Nejat et al., 2016). For instance, antiviral plant immune responses may interact with responses to additional environmental changes through cross-talks among hormonal pathways (Alazem and Lin, 2015; Nejat et al., 2016).

On the one hand, it has been shown that heat, drought or salt stress enhance plant susceptibility to pathogens (Atkinson et al., 2013; Kissoudis et al., 2015). On the another hand, viruses can enhance the ability of plants to counteract abiotic stresses by inducing drought or cold tolerance (Xu et al., 2008; Hily et al., 2016). Although the mechanisms involved are not clear, the authors evoked the increase of potential osmoprotectants in virus-infected plants (Xu et al., 2008). In the case of *Cucumber mosaic virus* (CMV), drought tolerance in *Arabidopsis thaliana* is triggered by the 2b viral RNA silencing suppressor protein, a viral protein interfering with abscisic acid-mediated plant signaling (Westwood et al., 2013). It has been speculated that this effect of 2b may ultimately serve viruses by aiding host plants to survive periods of environmental stress (Westwood et al., 2013).

Virus transmission is a key epidemiological parameter for which most plant viruses rely on arthropods vectors (Bragard et al., 2013). Impacts of abiotic stresses on virus spread have long focused on the vector biology (e.g., developmental time, longevity, fecundity, migration) and ecology (Nancarrow et al., 2014; Davis et al., 2015). While most of these studies speculated on a possible impact of environmental changes on the rate of virus transmission, direct experimental support was only brought very recently (Dáder et al., 2016; Nachappa et al., 2016; van Munster et al., 2017; Yvon et al., 2017). Because some studies suggest that transmission should be positively correlated to virulence, due to their shared relationship with viral accumulation (Alizon et al., 2009; Froissart et al., 2010) and that the environment may change viral transmission rate, abiotic stresses may also affect the relationship between these epidemiological parameters. Unfortunately, the plant’s surrounding environment is most often ignored when studying such epidemiological correlations (Fraile and García-Arenal, 2016).

In the present study, we monitored the effect of WD on various important viral life traits, such as viral accumulation, virulence and transmission. In order to explore the genetic variability of these traits and their relationships we selected nine wild accessions of *A. thaliana* (L.) Heynh (*Brassicaceae*) with known contrasted responses to WD (Rymaszewski et al., 2017). All accessions were infected with the *Cauliflower mosaic virus* (CaMV; *Caulimoviridae*), a non-circulative virus transmitted by aphids, and grown under strictly controlled environmental conditions in the high throughput phenotyping platform PHENOPSIS (Granier et al., 2006). Interestingly, our results suggest that the perturbing effects of WD on plant growth traits can change the trade-off between virus accumulation, virulence and transmission rate.

## Materials and Methods

### Plant Material and Growth Conditions

**FIGURE 1:**
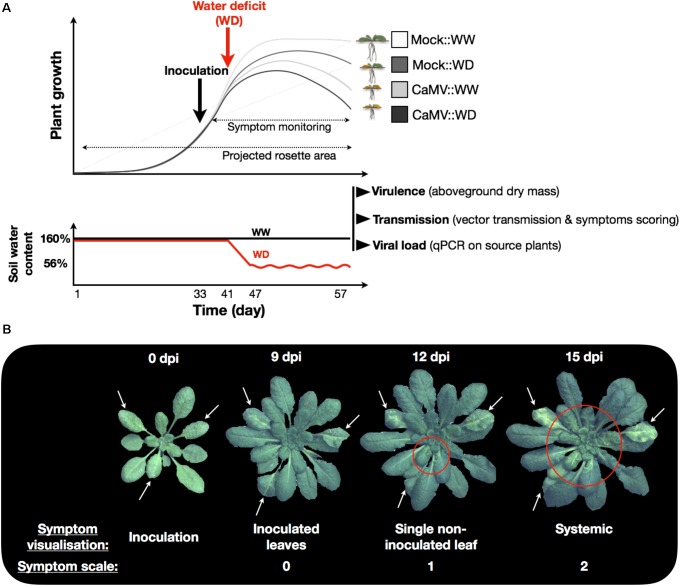
Schematic representation of the experiment and timing of measurements. **(A)** Plant inoculation (mock- or CaMV; black arrow) was realized 33 days after germination and corresponds to the beginning of symptom scoring. One week later, soil water deficit treatment (WD; red arrow) was applied by stopping watering. Mean soil water content of individual pots during the course of the experiment is represented in the second panel. Soil relative water content was maintained at 160% in the control treatment (WW; black line) while stopping irrigation in the WD treatment brought the soil water content to 56% after 7 days and was maintained to this value by an adequate water supply (WD; red line). At the end of the experiment (day 57), viral traits (virulence, transmission efficiency and viral accumulation in source plants) were estimated. **(B)** Symptom monitoring was performed daily (from 9 to 25 dpi) on CaMV-infected:WW (*n* = 20) and CaMV-infected:WD (*n* = 20) plants. Symptoms were scaled 0, 1, or 2 for no symptom (symptom on inoculated leaves), presence of symptoms on a single non-inoculated leaf, or systemic symptoms, respectively.

We selected nine natural accessions of *A. thaliana* (Mr-0, Col-0, Ct-1, Sha, Cvi-0, Mt-0, Bay-0, Ler-1 and Est-1) based on their contrasted responses to drought (Rymaszewski et al., 2017). Three to five seeds were sown at soil surface in 225 ml pots filled with a 30:70 (v/v) mixture of clay and organic compost (Substrate SP 15% KLASMANN). Soil water content was estimated for each pot before sowing, as previously described (Granier et al., 2006). Subsequent changes in pot weight were attributed to change in water status. Soil surface was moistened with a modified one-tenth strength Hoagland solution, and pots were placed in the PHENOPSIS growth chamber (Granier et al., 2006) in the dark for 2 days at 12°C air temperature and 70% air relative humidity. Pots were dampened with sprayed deionized water three times a day until germination. During germination phase (7 days), plants were cultivated under 8 h day length (200 μmol m^-2^ s^-1^ photosynthetic photon flux density, at plant height), air temperature was set to 20°C, and air relative humidity was adjusted in order to maintain constant water vapor pressure deficit (VPD) at 0.6 kPa. Then, plants were thinned to one plant per pot and grown at 21/18°C day/night while VPD was set at 0.75 kPa. Each pot was daily weighed and watered with the modified Hoagland solution to reach the target soil relative water content (RWC_soil_). RWC_soil_ was maintained at 1.6 g H_2_O g^-1^ dry soil until application of the WD treatment. One week before the application of the WD treatment, watering was done with deionized water until the end of the experiment (**Figure 1A**).

### Virus Purification and Plant Inoculation

The CaMV isolate Cabb B-JI (Delseny and Hull, 1983) efficiently transmitted following a non-circulative strategy by the aphid species *Myzus persicae*, was used in this study. Virus particles were purified from CaMV-infected *Brassica rapa* cv. “Just Right” (turnip) plants according to Hull and Shepherd (1976). The quality and the quantity of purified virus were assessed by polyacrylamide gel electrophoresis under denaturing conditions (12% SDS-PAGE) and by spectrometric measurements at 230, 260, and 280 nm (NanoDrop 2000 spectrophotometer). Virus concentration was estimated by spectrometry using the formula described by Hull and Shepherd (1976).

One-month-old *A. thaliana* plants (20 plants per accession per treatment) were mechanically inoculated as previously described (Blanc et al., 1993) with a mix containing CaMV-infected turnip extract enriched with the virus purification. Briefly, CaMV-infected turnip extract was prepared from 1 g of infected leaf material [leaves presenting systemic symptoms collected at 21 days post inoculation (dpi)] grinded in 1 ml of distilled water. Purified CaMV particles were then added to this mix at a final concentration of 0.2 mg ml^-1^ to optimize infection success. For each inoculated plant, three leaves of median rank in the rosette were rubbed with a small pestle soaked in the solution described above. Control group (eight plants per accession and per watering treatment) was mock-inoculated in a similar way to mimic the wound induced by mechanical inoculation. Mock-inoculation was performed with a mix containing non-infected plant extract and the buffer used for virus purification (100 mM Tris-HCl, 2.5 mM MgCl_2_, pH 7). All plants were inoculated in a random order, independent of their accession and of the watering regime. In summary, four conditions representing mock-inoculated:WW (*n* = 8), mock-inoculated:WD (*n* = 8), CaMV-infected:WW (*n* = 20) and CaMV-infected:WD (*n* = 20) plants per accession were analyzed.

### Water Deficit Treatment

One week after inoculation, corresponding to the approximate time of appearance of the first symptoms, irrigation of half of the CaMV- and mock-inoculated plants was stopped to reach the WD treatment at 0.56 H_2_O g^-1^ dry soil (this value was reached after 7 days of water deprivation). RWC_soil_ was then maintained to this value until the end of the experiment. In the well-watered treatment (WW) RWC_soil_ was maintained at 1.6 g H_2_O g^-1^ dry soil (**Figure 1A**).

### Measurement of Plant Traits and Symptoms Development

Projected rosette area (7–10 plants per accession and treatment) was estimated from automated daily pictures using a semi-automatic procedure developed in the image analysis environment Image J (Research Services National Institute of Mental Health, Bethesda, MD, United States) and downloadable on the PHENOPSIS web site (Fabre et al., 2011). For each accession and watering treatment (WW and WD), four mock-inoculated and ten CaMV-infected individual plants were harvested 25 dpi and individual aboveground dry mass was determined after 5 days at 60°C.

Symptom monitoring was performed daily (from 9 to 25 dpi) on CaMV-infected:WW (n = 20) and CaMV-infected:WD (n = 20) plants for each accession. The symptoms were scaled 0, 1, or 2 for no symptom, presence of symptom on a single non-inoculated leaf, or systemic symptoms, respectively (see illustration on **Figure 1B**). Time of symptoms appearance, rate of systemic spread, and maximum proportion of infected plants were then calculated from logistic regressions fitted to these observations.

### Aphid Rearing

The colony of the aphid-vector species *M. persicae*, collected over 30 years ago in the south of France was maintained on eggplants (*Solanum melongena*) in insect-proof cages, in a growth chamber at 23/18°C with a photoperiod of 14/10 h (day/night), in conditions ensuring clonal reproduction. Aphids were transferred to new cages and to new non-infested host plants (*Solanum melongena)* every 2 weeks, in order to avoid overcrowding and induction of the development of winged morphs.

### Aphid Transmission Assays

Transmission efficiency of CaMV was assessed at 25 dpi (Supplementary Figure S1). Batches of 20 *M. persicae* larvae (L2-L4 instars) were starved for 1 h before being transferred at the rosette center of a source plant for virus acquisition. Ten symptomatic source plants were used per accession and watering treatment. When aphids stopped walking and inserted their stylets into the leaf surface, they were allowed to feed for a short 2-min period. Viruliferous aphids were then immediately collected in a Petri dish and individually transferred to 1-month-old Col-0 plantlets (test plants) grown under non-stressing conditions (one aphid per test plant; nine test plants per source plant). After an inoculation period of 3 h, aphids were eliminated by insecticide spray (0.2% Pirimor G). Test plants were then placed in a growth chamber with the same conditions of air humidity, temperature and light as source plants and maintained under non-stressing conditions. Symptoms of virus infection were recorded 21 days later by visual inspection on test plants, as previously reported (Doumayrou et al., 2013; van Munster et al., 2017) and virus transmission rate was then calculated. After transmission assays, three leaves were randomly collected on each source plants and stored at -80°C for further nucleic acid extraction and quantification of the virus accumulation.

### Plant DNA Extraction

Total DNA from CaMV-infected leaf samples (pool of the three leaves collected per plant) was extracted according to a modified Edwards protocol (Edwards et al., 1991) with an additional washing step with 70% ethanol (10 biological replicates per accession and treatment). DNA was resuspended in 50 μl of distilled water, and 10-fold dilutions were used as qPCR templates. The quality and quantity of the extracted total nucleic acid were assessed by spectroscopic measurements at 230, 260, and 280 nm (NanoDrop 2000 spectrophotometer).

### DNA Quantification by qPCR

DNA quantification (10 biological replicates per accession and treatment, Supplementary Figure S1) was performed as duplicated qPCR in 384-well optical plates using the LightCycler FastStart DNA Master Plus SYBRGreen I kit (Roche) in a LightCycler 480 (Roche) thermocycler according to the manufacturer’s instructions. Specific primers designed for the quantification of CaMV genome (Ca4443-F: 5′-GACCTAAAAGTCATCAAGCCCA-3′ and Ca4557-R: 5′-TAGCTTTGTAGTTGACTACCATACG-3′) and *A. thaliana* ubiquitin-conjugating enzyme 21 gene (UBC21; UBC21_At_F: 5′-TGCAACCTCCTCAAGTTCGA-3′ and UBC21_At_R: 5′-GCAGGACTCCAAGCATTCTT-3′) were used at a final concentration of 0.3 μM. All qPCR reactions were performed with 40 cycles (95°C for 15 s, 62°C for 15 s and 72°C for 15 s) after an initial step at 95°C for 10 min. The qPCR data were analyzed with the LinReg PCR program to account for the efficiency of every single PCR reactions (Ruijter et al., 2009). The absolute initial viral concentration in *A. thaliana* plants, expressed in arbitrary fluorescence units (N_0_ CaMV) was divided by that of *A. thaliana UBC21* gene (N_0_ UBC21; Genbank accession DQ027035), in order to normalize the amount of plant material analyzed in all samples.

### Data Analyses

For each accession, the effects of the treatments on aboveground dry mass, transmission rate and viral load were analyzed by non-parametric Kruskal–Wallis tests. Time of symptoms appearance, rate of systemic spread, and maximum proportion of infected plants were extracted for each accession and watering treatment from logistic regression using the equation A/(1+exp ((4*μ/A)*(λ−t)+2)), where A is the maximum rate of infection, *λ* is the time necessary for the appearance of a systemic symptom on a non-inoculated leaf and *μ* is the time necessary to detect systemic symptoms on the full plant. The effect of watering on transmission rate was tested in a generalized linear model (glm) model with the binomial link function. Response ratios of aboveground plant dry mass (the ratios of mean outcome in the experimental group to that in the control group) were used to quantify the response of each genotype to watering and viral infection (i.e., virulence). We tested the significance of the relationships between epidemiological parameters with the Spearman’s rank correlation test.

All analyzes were performed in the programming environment R (R Core Team, 2017). Kruskal–Wallis tests were performed using the corresponding function in AGRICOLAE package. Bootstrapped 95% confidence intervals (CI) of mean trait values were computed following the *mean_cl_boot* procedure of the HMISC package. Non-linear models were fitted using the *nls* function and 95% confidence intervals for the parameters of fitted models were computed with *confint* function of the package MASS. Generalized linear models were tested using the *glm* function of the STAT package. Mean response ratios and corresponding 95% confidence intervals were calculated using *sci.ratio* of the MRATIO package.

## Results

### Systemic Spread Varies Between *A. thaliana* Accessions and, in Some Accessions, It Is Conditioned by Watering Treatment

**FIGURE 2:**
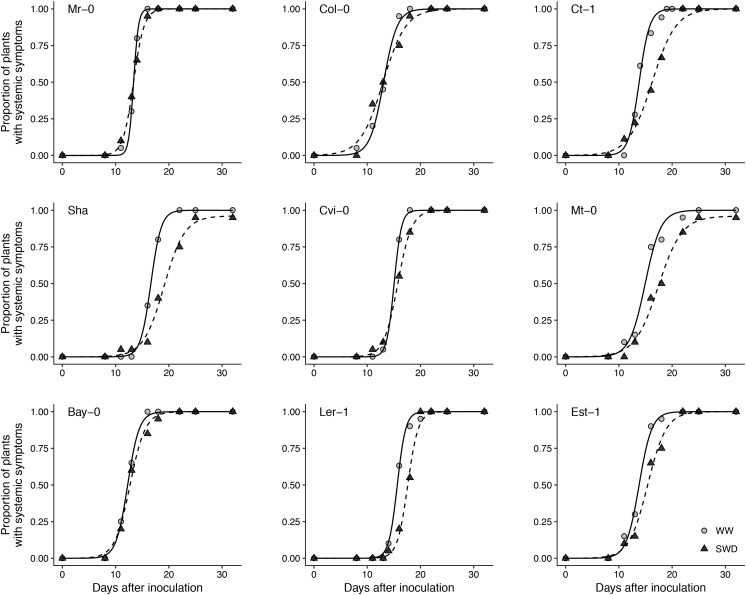
Symptom dynamics in nine *A. thaliana* accessions inoculated with CaMV and grown under two watering conditions. Each panel represents one accession grown under well-watered (gray circles) and water deficit (black circles), respectively. Points are means of the proportion of plants with systemic symptoms (*n* = 20 plants per accession and watering treatment). Curves are logistic fitting following equation A/(1+exp ((4*μ/A)*(λ−t)+2)), where *t* is the number of days after inoculation, A is the maximum rate of infection, *λ* is the time necessary for the appearance of symptoms on a non-inoculated leaf and *μ* is the time required to visualize systemic symptoms.

**FIGURE 3:**
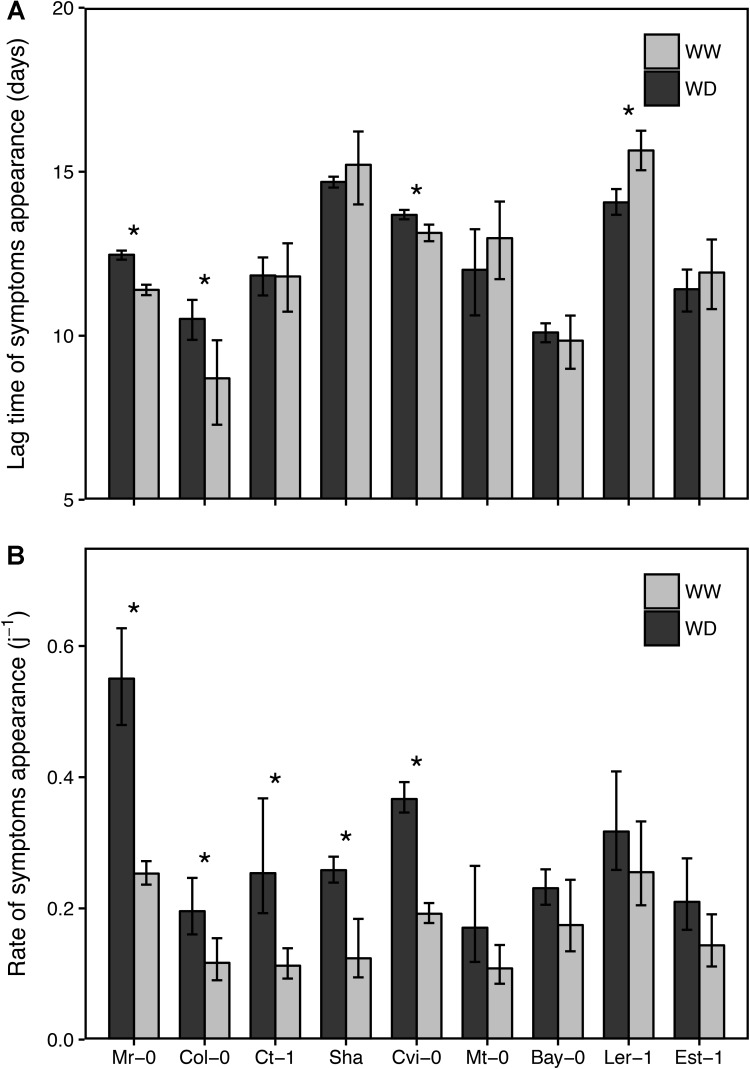
Lag time and rate of CaMV symptoms appearance in nine *A. thaliana* accessions grown under two watering conditions. Bars and error bars are means and bootstrapped 95% confidence intervals of lag time **(A)** and rate **(B)** of symptoms appearance extracted from sigmoidal curve fitting of symptom dynamics (see **Figure 1**) under well-watered (gray bars) and water deficit (black bars), respectively. Stars indicate significant difference between watering treatment for each accession (nonparametric Kruskal–Wallis tests; *P* < 0.05). Accessions are ordered according to increasing tolerance to WD.

CaMV isolate Cabb B-JI successfully infected plants of all *A. thaliana* accessions selected. Symptoms, i.e., chlorotic lesions and vein-clearing of rosette leaves, were similar across accessions though their timing of appearance and intensity was greatly variable as detailed below. The proportion of CaMV-inoculated plants showing characteristic virus symptoms 25 dpi varied from 96 to 100% across accessions whatever the soil watering treatment (**Figure 2**). In WW condition, the mean time of systemic symptoms appearance on the first non-inoculated leaf (±95% CI) was 12.3 (±1.9) dpi (**Figure 3A**). However, lag time of systemic symptoms varied significantly between accessions from 10 (±0.3) dpi in Bay-0 to 14.7 (±0.2) dpi in Sha. Lag time did not change in response to WD in five accessions whereas it was significantly lower (i.e., faster appearance of firsts symptoms) in three accessions and higher in one accession (**Figure 3A**). For all accessions, the rate of systemic spread was lower under WD than in WW (**Figure 3B**). In particular, it was significantly reduced for Mr-0, Col-0, Ct-1, Sha and Cvi-0 (**Figure 3B**).

### Plant Growth Response to Water Deficit and Viral Infection Vary Across Natural Accessions

**FIGURE 4:**
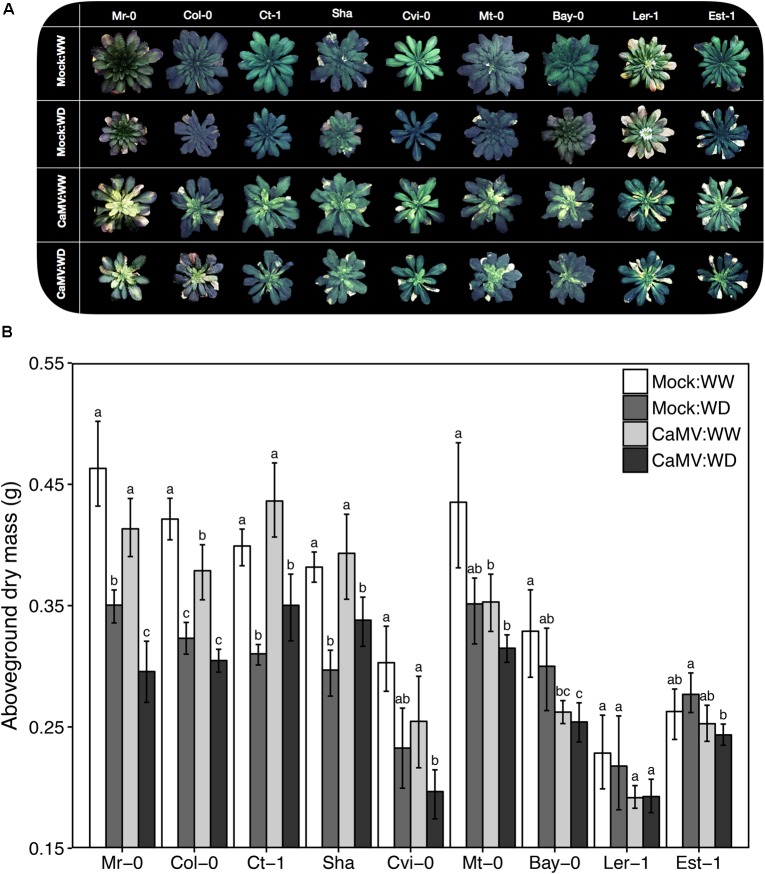
Illustrative photographs of the rosettes and aboveground dry mass of nine *A. thaliana* accessions inoculated with CaMV and grown under two watering conditions. **(A)** Illustrative photographs of the rosettes of nine *A. thaliana* accessions (Mr-0, Col-0, Ct-1, Sha, Cvi-0, Mt-0, Bay-0, Ler-1 and Est-1) inoculated with a mock treatment or with CaMV and grown under well-watered (WW) or water deficit (WD) conditions. All photographs were taken 24 days postinoculation (dpi), 1 day before the transmission test. **(B)** Aboveground dry mass of nine *A. thaliana* accessions. Bars and error bars are means ± bootstrapped 95% confidence intervals of aboveground dry mass 25 dpi and 19 days after the start of water deficit treatment (WD). Mock-inoculated:WW (white bars, *n* = 8), mock-inoculated:WD (dark gray bars, *n* = 8), CaMV-infected:WW (light gray bars, *n* = 20) and CaMV-infected:WD (black bars, *n* = 20) conditions. Different letters indicate a significant difference between treatments for each accession (nonparametric Kruskal–Wallis tests; *P* < 0.05). Error bars were not used to interpret the results. Accessions are ordered according to increasing tolerance to WD.

We selected nine natural accessions of *A. thaliana* (Mr-0, Col-0, Ct-1, Sha, Cvi-0, Mt-0, Bay-0, Ler-1 and Est-1) based on their contrasted responses to drought (**Figure 4A**). Plant aboveground dry mass (±SD) ranged from 0.21 (±0.02) g (Ler-1) to 0.46 (± 0.04) g (Mr-0) under WW condition (**Figure 4B**). As determined 19 days after the start of the treatment, WD reduced aboveground dry mass production (20–25% reduction) in the four accessions the less tolerant to WD: Mr-0, Col-0, Ct-1 and Sha. Growth reduction due to WD was marginally significant in Cvi-0 (*P* = 0.083), and not significant in Mt-0, Bay-0, Ler-1 and Est-1, which are accessions the most tolerant to WD (**Figure 4B**).

As determined at 25 dpi, CaMV virulence under WW, calculated as CaMV-infected:WW/mock-inoculated:WW for aboveground biomass, varied significantly among accessions. CaMV infection significantly reduced aboveground dry mass (10–21% reduction) in all accessions but Ct-1, Sha and Est-1 (**Figure 4B**). In particular, aboveground dry mass of CaMV-infected plants was significantly reduced in Bay-0 (*P* = 0.0072), Col-0 (*P* = 0.048) and Mt-0 (*P* = 0.047).

In general, the combination of WD and viral infection tended to be more deleterious than each of the two stresses taken separately. However, plant responses differed widely between accessions. For example, WD and CaMV infection combination did not have a significant effect on aboveground mass of Est-1 and Ler-1 compared to the mock-inoculated:WW condition, whereas a 13–40% significant reduction was found in five other accessions (*P* < 0.05; **Figure 4B**). However, in two accessions, Sha and Ct-1, stress combination was less severe than the effect of WD alone although it was no significant (Sha: *P* = 0.15 and Ct-1: *P* = 0.20; **Figure 4B**). Similar trends were observed for the projected rosette area since this trait was highly significantly correlated to aboveground dry mass (*R^2^* = 0.68, *P* < 0.001; Supplementary Figure S2). Response ratios of aboveground biomass of each accession and for the different combinations of treatment are presented in Supplementary Figure S3.

### Variation of Viral Load and Transmission Rate Under WD Is Dependent on the Accession

**FIGURE 5:**
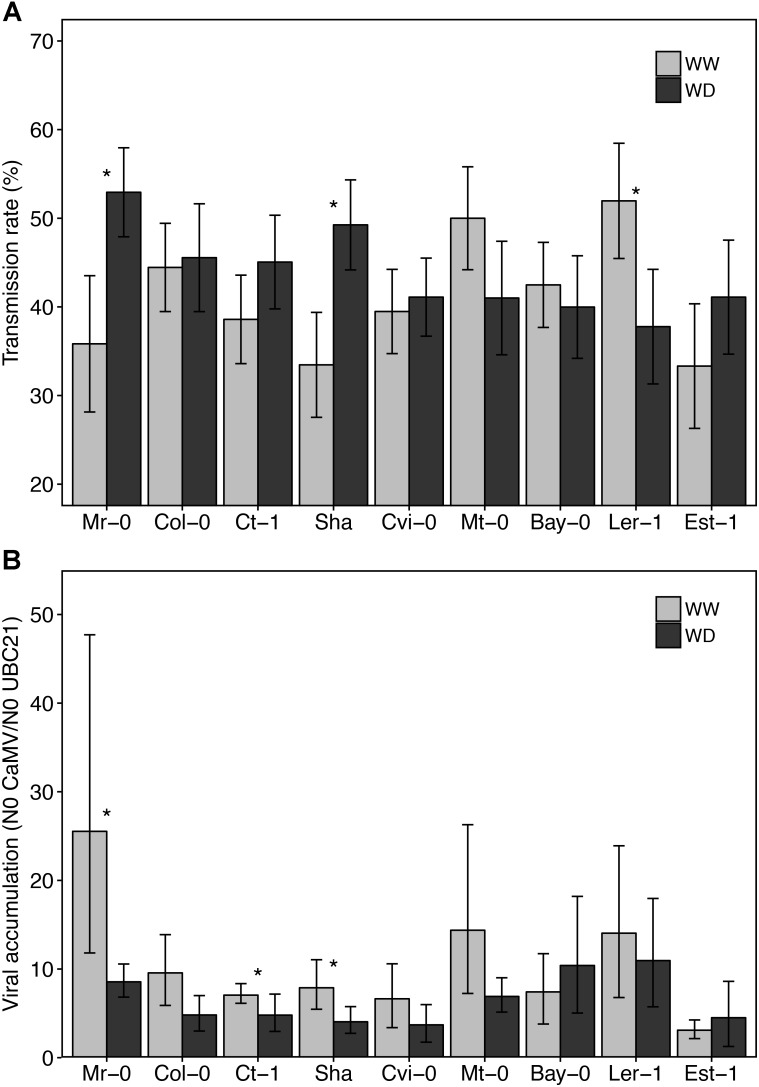
CaMV transmission rate and accumulation in nine *A. thaliana* accessions grown under two watering conditions. Transmission rate **(A)**, i.e., mean proportion of infected test plants (*n* = 9 per source plant) and viral load **(B)** of source plants (*n* = 10) of each accession grown under well-watered (gray bars) and water deficit (black bars), 25 dpi and 19 days after the start of water deficit treatment, respectively. Stars indicate a statistically significant difference between watering treatment for each accession (nonparametric Kruskal–Wallis tests; *P* < 0.05). Error bars represent bootstrapped 95% confidence intervals but were not used to interpret the results. Accessions are ordered according to increasing tolerance to WD.

Preliminary experiments showed that CaMV transmission rate (from a virus donor infected plant) did not vary significantly according to the identity of the accession used as test plant (receptor plant) in transmission experiments (data not shown). For practical reasons, we therefore used Col-0 as test plants in subsequent transmission assays. Whatever the watering treatment and accession, transmission rate varied from 30 to 57% (**Figure 5A**). Surprisingly, while in most accessions WD did not significantly affect the transmission rate or marginally reduced it (Ler-1, from 55 to 38%, *P* < 0.10), we observed a significant increase of transmission rate in Mr-0 and Sha (**Figure 5A**). In these two latter accessions transmission rate increased from 35–51% to 38–57%, respectively (*P* < 0.05). Noteworthy, a similar trend was observed for Ct-1 and Est-1 (**Figure 5A**).

Viral load, i.e., CaMV accumulation in source plants, significantly decreased when plants were grown under WD compared to WW condition for Mr-0, Ct-1, and Sha (10–25% reduction, *P* < 0.05), and tended to decrease in Col-0, Cvi-0, Mt-0 and Ler-1 (**Figure 5B**). No correlation was found between viral load and transmission rate across accessions whatever the watering condition (Spearman’s *r* = 0.40, *P* = 0.29 in WW; Spearman’s *r* = 0.40, *P* = 0.17 in WD).

### Water Deficit Alters the Relationship Between Transmission Rate and Virulence

**FIGURE 6:**
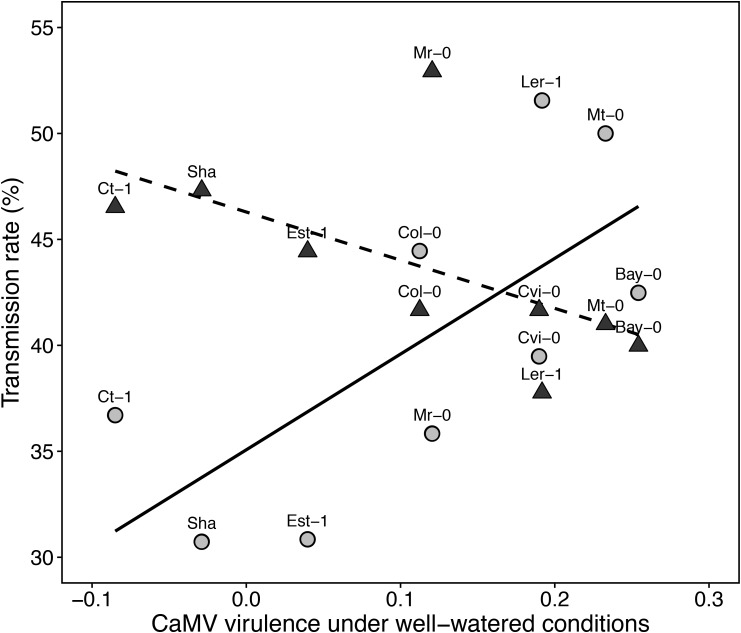
Relationship between virulence and transmission rate of CaMV in nine *A. thaliana* accessions. Each labeled point represents CaMV virulence under well-watered and transmission rate under well-watered (gray circles) and water deficit (black triangles) for each accession. Lines represent linear regressions under well-watered (solid line; Spearman’s *r* = 0.68, *P* = 0.05) and water deficit (dashed line; Spearman’s *r* = -0.75, *P* = 0.019), respectively.

A significant positive correlation between CaMV virulence (i.e., CaMV-infected:WW/mock-inoculated:WW response ratio of aboveground dry mass) and transmission rate was found under WW (Spearman’s *r* = 0.68, *P* < 0.05; **Figure 6**). In other words, transmission rate was higher in accessions the most susceptible to CaMV infection in terms of aboveground mass. Under WD, transmission rate increased in accessions that were the most tolerant to CaMV while the reverse trend was observed in accessions more susceptible to CaMV. As a result, a significant negative correlation between transmission rate under WD and CaMV virulence was found (Spearman’s *r* = -0.75, *P* < 0.02; **Figure 6**). The relationship between virulence and transmission was distorted regardless of the response ratio calculated for virulence (Supplementary Figures S4, S5).

## Discussion

We investigated plant responses to simultaneous exposure to WD and infection with one isolate of the CaMV in nine wild accessions of *A. thaliana* (L.) Heynh. We tested the hypothesis that perturbing effects of WD on plant growth traits would lead to changes in key epidemiological parameters such as systemic spread, virulence, viral load and transmission rate. Our results showed that under WD, viral infection spread into the plant was slower compared to WW treatment. Furthermore, WD had contrasted effects on CaMV transmission rate and viral load among *A. thaliana* accessions. Under WW, transmission rate tended to increase with the susceptibility of the accession to CaMV. Under WD, transmission rate and susceptibility were negatively correlated.

Most plant viruses move in a systemic way within the host plant (Matthews, 1991; Leisner et al., 1993). After inoculation by an aphid vector or by mechanical inoculation like in the present study, CaMV re-initiate infections in different and distant tissues, and such viral spread guarantees its survival (Schoelz et al., 2015). Virus spread within the host plant happens through cell-to-cell movement (via plasmodesmata) and long-distance movement mainly through phloem vessels (Matthews, 1991; Stavolone et al., 2005). It is known that the time at which viruses move out of the inoculated leaf into the rest of the plant varies widely depending on factors such as host and virus species, age of the host, method of inoculation and abiotic constraints (Jensen, 1973; Ismail and Milner, 1988). In this study, we showed that the lag time to symptoms appearance (the mean time for symptoms to appear in the first non-inoculated leaf) and rate of systemic spread (the time required for all the plants of an accession to exhibit systemic symptoms) of CaMV was affected by accession identity and WD. Previous studies have already shown that inherent differences in development, particularly flowering phenology, and growth, can affect the dynamics of viral infection across accessions (Leisner et al., 1993). Virus spread is also influenced by the flow of metabolites in the plant (Bennett, 1940). Since WD may affect the relationship between carbon availability and sink organ growth (Muller et al., 2011), it can be assumed that long-distance transport of viral particles through phloem will also be affected, leading to slower systemic movement (Córdoba et al., 1991; Leisner et al., 1993; Leisner and Howell, 1993). Here, correlated with the negative effects of WD on plant growth and development we observed a significant reduction in the rate of systemic symptoms appearance in the most WD-sensitive accessions such as Mr-0 and Ct-1. This reinforces the idea of a strong interaction between growth, phenology and viral infection dynamics. These observations are confirmed by the negative correlation between time or rate of systemic spread and tolerance to WD but also with the time of appearance of flowering buds (i.e., bolting) among accessions (not shown).

Regarding the impact of each independent stress on growth, CaMV infection had a generally lower negative effect on plant growth than WD since the virulence of the viral strain Cabb B-JI on vegetative growth was not too deleterious for most accessions studied. The effect of double stress was even more detrimental to rosette growth compared to control conditions. There was no correlation between tolerance to WD and tolerance to CaMV infection. Despite several reports on virus capacity to improve plant tolerance to abiotic stresses (Xu et al., 2008; Westwood et al., 2013), we could not find any significant positive effect of virus infection on plant tolerance to WD.

The success of transmission from infected to healthy host plants is crucial for the survival of all plant viruses. Interestingly, we showed that a WD triggered a significant increase of transmission rate in Sha and Mr-0, two accessions among the most sensitive to WD as previously described (Rymaszewski et al., 2017). These results are consistent with the earlier report of increased transmission of CaMV and *Turnip mosaic virus* (TuMV) from *B. rapa* plants submitted to WD (van Munster et al., 2017). However, the variation of transmission rate under abiotic stress depends on the plant-virus-vector pathosystem and on the type of abiotic stress (Dáder et al., 2016; Nachappa et al., 2016; Yvon et al., 2017). It has been anticipated that abiotic stresses can impact multiple steps of the intricate plant–virus–vector interactions and so modify the transmission rates in many different ways (Mauck et al., 2012; Blanc and Michalakis, 2016). For example, it was demonstrated that the CaMV can ‘sense’ the aphid feeding activity and immediately produce transmissible morphs, and that this viral “behavior” is also triggered by some abiotic stresses (Martinière et al., 2013). This remarkable phenomenon has been designated ‘transmission activation’ (Martinière et al., 2013). It is most probably triggered by activation of plant defense pathways against aphid attacks, and that some abiotic stresses induce similar effects is likely due to partially overlapping pathways (Kiegle et al., 2000; Fujita et al., 2006; Suzuki et al., 2014; Pandey et al., 2015). In our study, we revealed that most WD-sensitive accessions have an increased CaMV transmission rate under WD, despite a reduced virus accumulation. This surprising observation might reflect a higher induction of signaling/defense pathways than in others accessions, and thus potentially, a stronger ‘transmission activation’ of the CaMV.

Virus accumulation in source plants was evaluated as a potential explanatory factor of the altered transmission efficiency. Indeed, it has been reported that abiotic stresses modify viral load of several viruses, such as *Tobacco mosaic virus* and *Potato virus A*, within stressed host plants (Dorokhov et al., 2012; Suntio and Mäkinen, 2012). Moreover, a positive correlation between virus load and transmission efficiency has also been reported in some instances (Froissart et al., 2010; Doumayrou et al., 2013). In our study, we could not find any significant correlation between these two traits, i.e., a higher viral particles accumulation in the plant did not invariably correlate with a higher transmission. In addition to the phenomenon of the transmission activation discussed above, we also emphasize that many other unknown factors may be responsible for the observed altered transmission under water deficit condition. For example, feeding behavior of the aphid vector may differ on WD infected plants as a consequence of change in plant quality such as changes in carbon-to-nitrogen ratio (Gutiérrez et al., 2013; Prasch and Sonnewald, 2013; Traaaebicki et al., 2016) and/or morpho-anatomical changes (Carmo-Sousa et al., 2014; Dáder et al., 2017).

In our CaMV-Arabidopsis pathosystem, virulence, as measured by the response ratio of aboveground dry mass, was significantly positively and linearly correlated to transmission rate under WW. The positive relationship between basal virulence and transmission is in accordance with evolutionary expectations as well as other empirical observations (Asplen et al., 2012; Doumayrou et al., 2013; Alizon and Michalakis, 2015). The ‘trade-off hypothesis’ postulates that increased virulence of a parasite is a viable evolutionary strategy if and only if its costs (mortality of the host) are counterbalanced by an increased transmission efficiency (Pagán et al., 2007). For instance, a positive relationship but with a saturating trend between these two traits was found in the CaMV-*Brassica rapa* pathosystem by Doumayrou et al. (2013) when testing different natural CaMV isolates on one cultivar of *B. rapa*. Surprisingly, we showed that WD had a significant reversing effect on this relationship. Indeed, CaMV transmission rate from the most virus-tolerant *A. thaliana* accessions (Sha, Ct-1, Est-1) significantly increased under WD while it decreased or did not change in other accessions. To our knowledge, this is the first study showing an alteration of the relationship (here reversed) between viral tradeoffs due to a change in abiotic conditions (Fraile and García-Arenal, 2016).

## Conclusion

Our results support the idea that optimal virulence of a given virus, as hypothesized under the transmission-virulence trade-off, is highly dependent on the environment and growth traits of the host. The multi-faceted relationships between virulence, viral load and transmissibility according to the environmental conditions experienced by the host will require further theoretical and experimental investigations. In particular, investigations concerning the behavior of the aphid (alteration of its behavior by the environment) but also on the implication of plant and virus genetic diversity. Especially if these relationships have to be incorporated into models of virus epidemiology under scenarios of climate changes.

## Author Contributions

SEB, MvM, SB, and DV conceptualized the study. SEB, MvM, MD, AB, CV-R, GR, MY, and DV performed the experiments. SEB, MvM, and DV analyzed the data and wrote the original draft. All coauthors edited and reviewed the final version of the paper.

## Conflict of Interest Statement

The authors declare that the research was conducted in the absence of any commercial or financial relationships that could be construed as a potential conflict of interest. The reviewer AG and handling Editor declared their shared affiliation.
