# Supplementary material for: Interactions Between Drought and Plant Genotype Change Epidemiological Traits of Cauliflower mosaic virus
Source: Front Plant Sci. 2018 May 24;9:703. doi: 10.3389/fpls.2018.00703 (PMC5976794; doi:10.3389/fpls.2018.00703)
Supplement: Supplementary file 1 [file Image_1.pdf]

Supplementary Material

Interactions between drought and plant genotype change epidemiological traits of *Cauliflower mosaic virus*

Sandy E. Bergès[1,2], Denis Vile[2*], Cecilia Vazquez-Rovere[2,3], Stéphane Blanc[1], Michel Yvon[1], Alexis Bediée[2], Gaëlle Rolland[2], Myriam Dauzat[2], Manuella van Munster[1*]

[1]BGPI, Univ Montpellier, CIRAD, INRA, Montpellier SupAgro, Montpellier, France.

[2]LEPSE, Univ Montpellier, INRA, Montpellier SupAgro, Montpellier, France

[3]LABINTEX-Europe, INTA, Montpellier, France

*Corresponding authors: manuella.van-munster@inra.fr; denis.vile@inra.fr

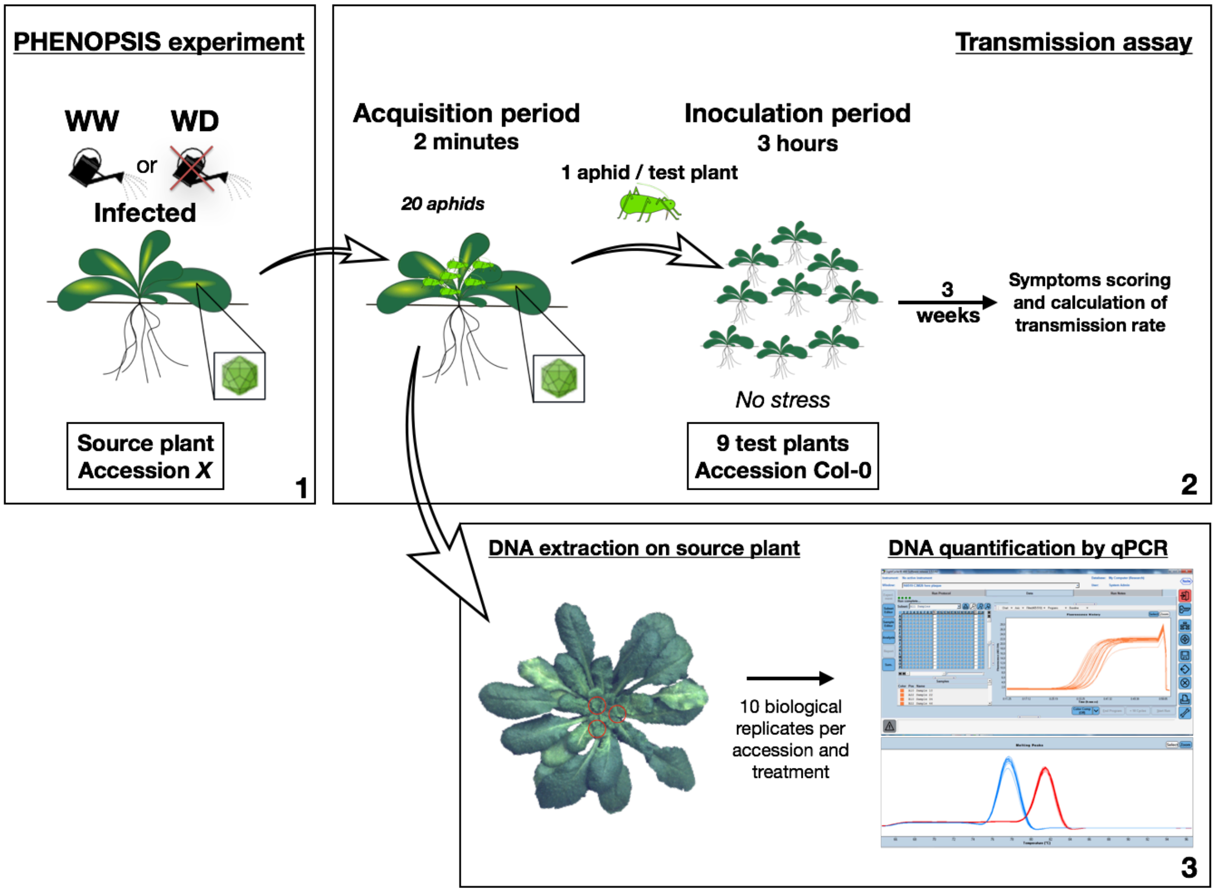

Figure S1. Experimental design for CaMV transmission assay and qPCR experiment. (1) Source plant development in PHENOPSIS platform under two water soil conditions, CaMV-WW (well-watered) or CaMV-WD (water deficit). **(2)** Transmission efficiency of CaMV was assessed at 25 dpi. Batches of 20 *M. persicae* larvae (l2-L4 instars) were transferred on the rosette center of a source plant for virus acquisition. Ten symptomatic source plants were tested per accession and watering treatment. When aphids stopped walking and inserted their stylets into the leaf surface, they were allowed to feed for a short two-minutes period. Viruliferous aphids were then immediately collected in a Petri dish and individually transferred to one-month-old Col-0 plantlets (test plants) grown under no stress conditions (one aphid per test plant; nine test plants per source plant). After an inoculation period of three hours, aphids were eliminated by insecticide spray (0.2% Pirimor G). Test plants were then placed in a growth

chamber with the same conditions of air humidity, temperature and light as source plants and maintained under no stress conditions. Symptoms of virus infection were recorded 21 days later by visual inspection on test plant and virus transmission rate was calculated. **(3)** After transmission assays, three leaves were randomly (red circles) collected on each source plants and stored at -80 °C for further nucleic acid extraction and quantification of virus accumulation by qPCR.

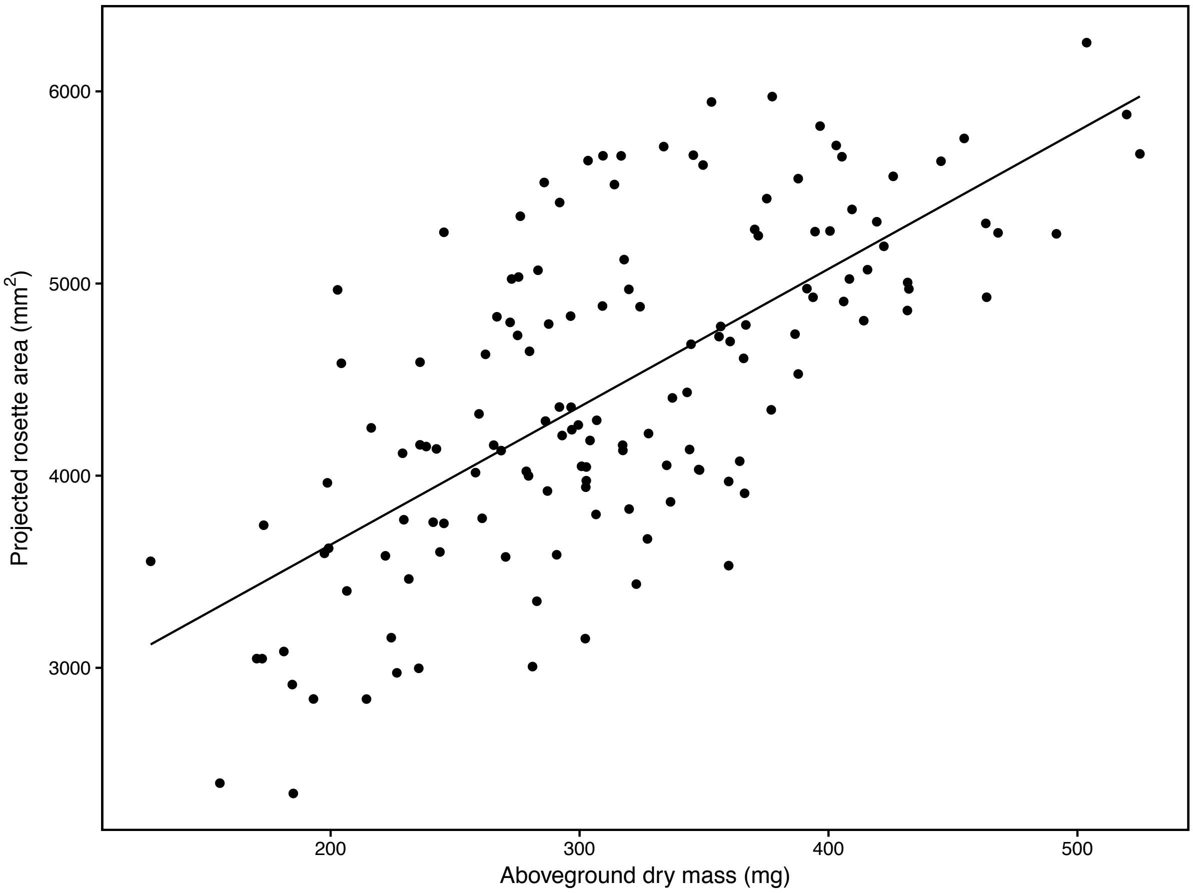

Figure S2. Relationship between aboveground dry mass (mg) and projected rosette area (mm^2). Linear regression includes all accessions regardless of water treatment or viral infection (R^2 = 0.68, P < 0.001).

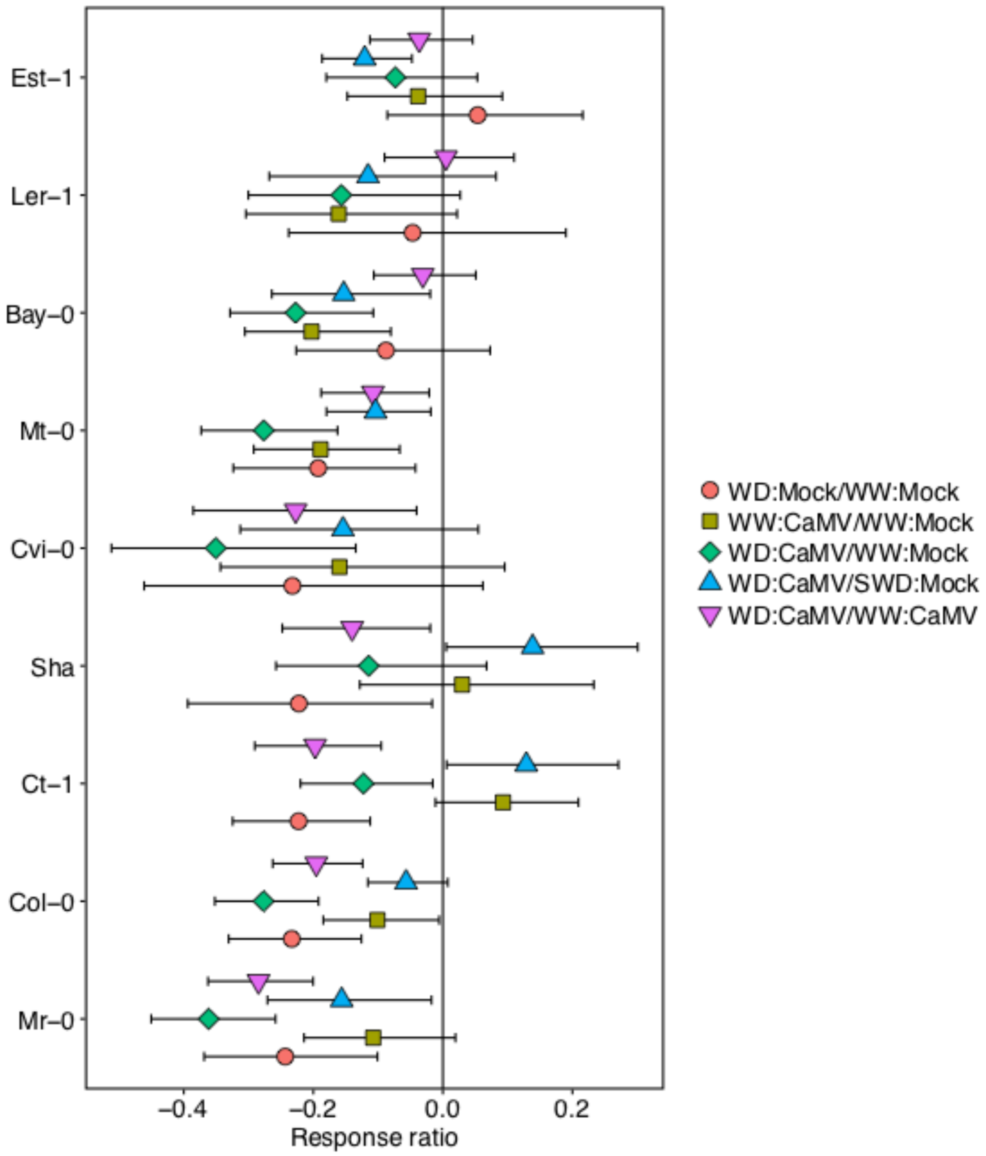

Figure S3. Response ratios of aboveground biomass for each accession. Mean response ratios of mock-inoculated plants to WD (red circle), CaMV-infection under WW conditions (green square), CaMV infection under WD compared to WW:mock-inoculated (green diamond), CaMV infection under WD compared to WD:mock-inoculation (blue triangle) and CaMV infection under WD compared to WW-CaMV inoculated (pink upside-down triangle). Mean response ratios are sorted by the less tolerant accession to WD at bottom to the most tolerant one on top, and are surrounded by 95% confidence intervals. Differences were significant when 95% confidence intervals did not overlap zero.

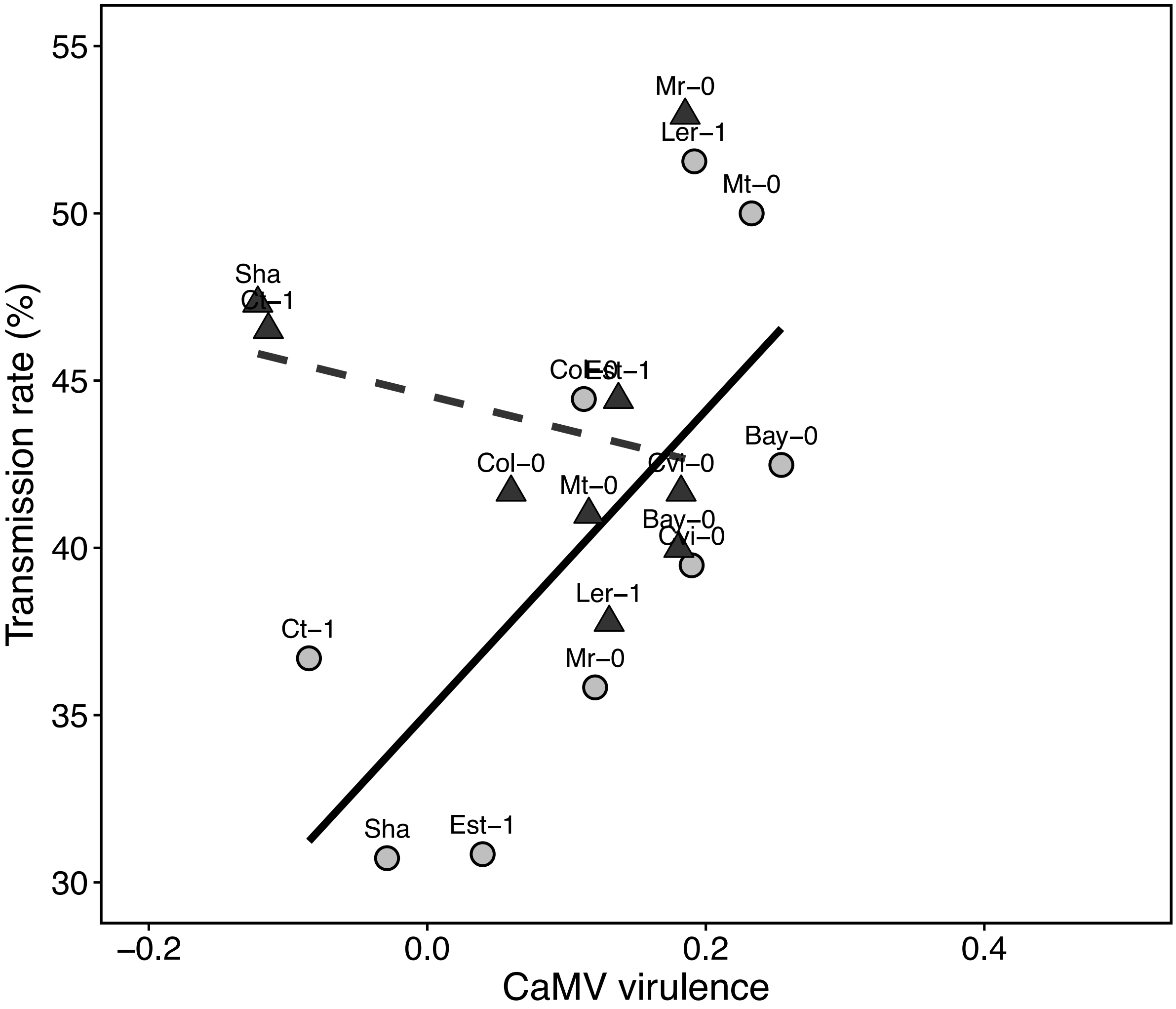

Figure S4. Relationship between virulence (response ratio of aboveground dry mass mock-inoculated under water deficit (WD)/CaMV-infected under WD condition and transmission rate of CaMV in nine *A. thaliana* accessions. Labelled points represent CaMV virulence calculated as the response ratio of aboveground dry mass mock-inoculated/CaMV-infected under well-watered (WW) condition, and CaMV transmission rate under WW (grey circles), or CaMV virulence calculated as the response ratio of aboveground dry mass mock-inoculated under WD/CaMV-infected under WD, and CaMV transmission rate under WD (black triangles) for each accession. Lines represent linear regressions under WW (solid line; $r_{\text{Spearman}} = 0.68$, $P = 0.05$) and SWD (dashed line; $r_{\text{Spearman}} =$, $P = 0.81$), respectively.

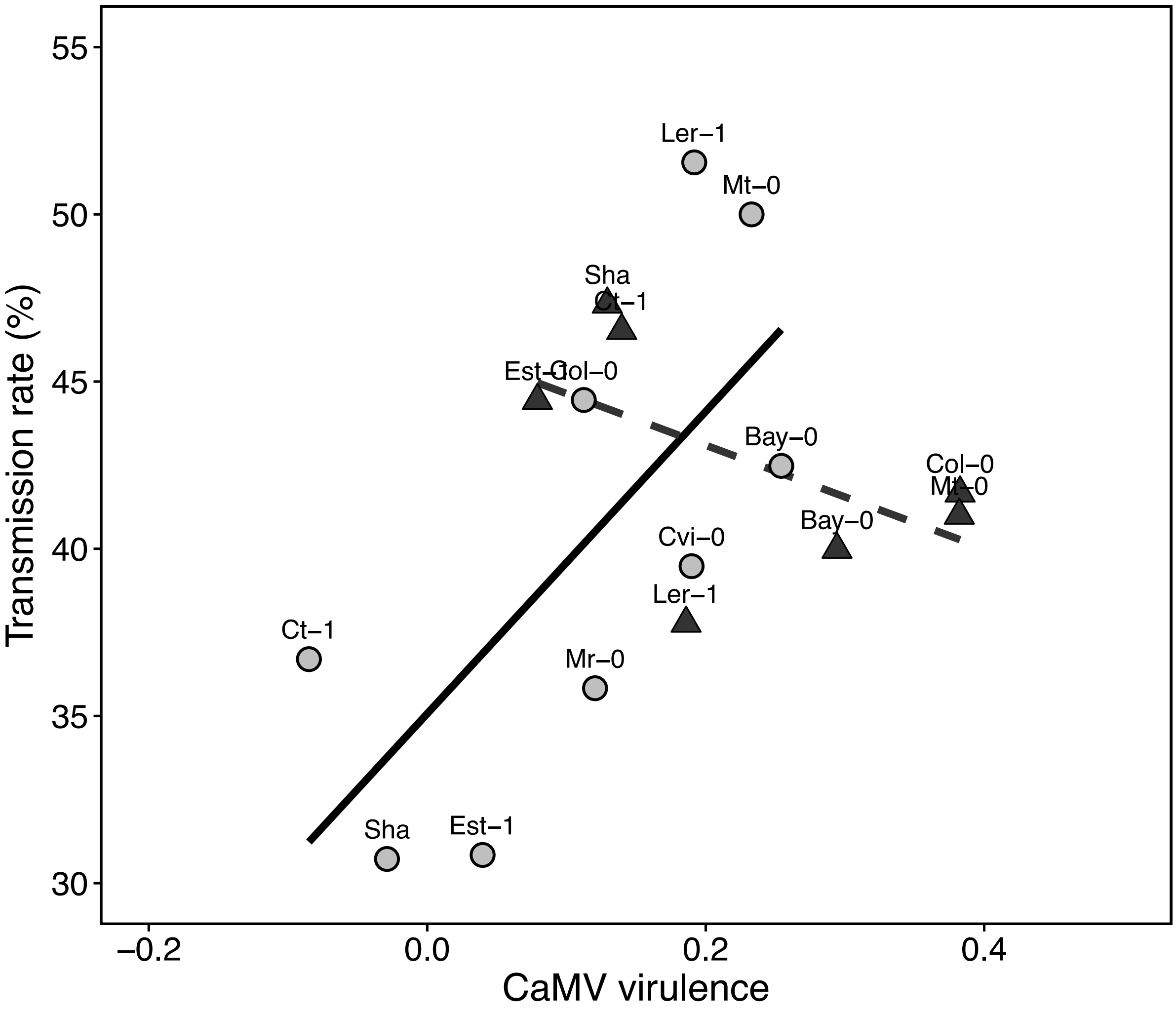

Figure

Figure S5. Relationship between virulence (the response ratio of aboveground dry mass mock-inoculated under WW/CaMV-infected under WD condition) and transmission rate of CaMV in nine *A. thaliana* accessions. Labelled points represent CaMV virulence calculated as the response ratio of aboveground dry mass mock-inoculated/CaMV-infected under well-watered (WW) condition, and CaMV transmission rate under WW (grey circles), or CaMV virulence calculated as the response ratio of aboveground dry mass mock-inoculated under WW/CaMV-infected under WD condition, and CaMV transmission rate under WD (black triangles) for each accession. Lines represent linear regressions under WW (solid line; r_{Spearman} = 0.68, P = 0.05) and WD (dashed line; r_{Spearman} =, P = 0.95), respectively.
